# Preservation of Basic Life Support Competencies Among Certified First Responders

**DOI:** 10.3390/healthcare14121654

**Published:** 2026-06-11

**Authors:** Igor Goričan, Andrej Šorgo, Matej Strnad

**Affiliations:** 1 General Staff, Slovenian Armed Forces, Ministry of Defence, Vojkova 55, 1000 Ljubljana, Slovenia; 2 Department of Emergency Medicine, Faculty of Medicine, University of Maribor, Taborska Ulica 8, 2000 Maribor, Slovenia; 3 Faculty of Health Sciences, Alma Mater Europea University, Slovenska Ulica 17, 2000 Maribor, Slovenia; 4 Department of Biology, Faculty of Natural Sciences and Mathematics, University of Maribor, Koroška Cesta 160, 2000 Maribor, Slovenia; 5 Prehospital Unit, Center for Emergency Medicine, Community Healthcare Center, Cesta Proletarskih Brigad 21, 2000 Maribor, Slovenia; 6 Emergency Department, University Medical Center Maribor, Ljubljanska Ulica 5, 2000 Maribor, Slovenia

**Keywords:** first responders, CPR, education, resuscitation, volunteers, training, certification, emergency responders, emergency, out-of-hospital cardiac arrest

## Abstract

**Background and Objectives:** Slovenia, like many other European nations, has introduced voluntary first responders to enhance survival rates in out-of-hospital cardiac arrests. In currently published research, no conclusive data exists on the optimal retraining interval, categorizing recommendations as expert opinion with limited reliability. **Materials and Methods:** Between 2019 and 2024, observational longitudinal research and a cross-sectional comparison was conducted in accordance with national guidelines on Slovenian newly certified (N = 342; 227 males, 112 females and three participants with missing or undetermined gender; mean age, 31.3 ± 12.2 years) and senior (N = 140; 105 males, 35 females; mean age, 38.0 ± 11.9 years) licensed first responders (LFRs) with 5–10 years of experience (median = 6.5, IQR 6–7). LFRs were reassessed for retention of skills and knowledge one year after previous certification. Additionally, each cohort was classified into groups according to the number of interventions they engaged in over the past year, and their retention of skills and knowledge was assessed. **Results:** During the initial year of service, no statistically significant decline in practical skills (median 53 [52–54] vs. 53 [50–54]; *p* = 0.059) or theoretical knowledge (median 10 [9–10] vs. 9 [9–10]; *p* = 0.458) was observed among newly certified LFRs. In contrast, senior LFRs demonstrated significantly lower practical skill scores prior to recertification compared with newly certified LFRs (median 51 [49–54] vs. 54 [52–55]; *p* < 0.001), whereas theoretical knowledge remained stable (median 9 [8.5–10] vs. 10 [9–10]; *p* = 0.091). Intervention frequency did not affect skill or knowledge retention among newly certified LFRs. However, senior LFRs who did not participate in any interventions during the certification period demonstrated significantly lower practical skill scores comparable to newly certified LFRs (median 49 [47–51] vs. 54 [52–55]; *p* < 0.001), whereas those who participated in at least one intervention showed no significantly lower scores (median 52 [50–54] vs. 54 [52–55]; *p* = 0.117). No significant reduction in theoretical knowledge was observed. Linear regression analysis demonstrated that participation in at least one intervention significantly predicted higher practical skill scores among senior LFRs (β = −1.11, *p* = 0.001), whereas no significant association was observed for theoretical knowledge retention or among newly certified LFRs. **Conclusions:** The initial training for Slovenian LFRs was found to be adequate. However, senior LFRs who did not participate in interventions demonstrated lower practical skill performance compared with newly certified LFRs. Further evaluation and different strategies for recertifying senior LFRs should be adopted, considering the number of interventions they have been involved in after last certification.

## 1. Introduction

Out-of-hospital cardiac arrest (OHCA) ranks among the leading causes of potentially avoidable fatalities, with inevitable outcomes if no action is taken. In both the United States and Europe, approximately 350,000 to 450,000 OHCAs occur annually [1,2]. A recent study established that the annual incidence of OHCA in Slovenia is 77.32 per 100,000 individuals, which corresponds to an approximate incidence rate of 1624 per year for Slovenia’s population of 2.1 million [3].

Although OHCA often occurs suddenly, a substantial proportion is related to potentially modifiable cardiovascular risk factors and underlying cardiac disease [4]. Preventive measures therefore include primary prevention of coronary artery disease and arrhythmia-related risk through control of hypertension, diabetes, dyslipidemia, obesity, smoking, and physical inactivity, as well as timely recognition and treatment of warning symptoms such as chest pain, dyspnea, palpitations, syncope, or unexplained collapse. The most common causes of OHCA include acute coronary syndromes, structural heart disease, and malignant ventricular arrhythmias, while non-cardiac causes such as respiratory failure, trauma, drowning, pulmonary embolism, intoxication, or electrolyte disturbances may also occur [5,6,7]. Clinically, cardiac arrest usually presents as sudden unresponsiveness, absent or abnormal breathing, and the absence of signs of circulation. Because survival depends on immediate recognition, early CPR, and rapid defibrillation, community education, AED availability, optimized EMS response, and organized first responder systems are essential components of OHCA prevention and post-event survival improvement [2,8].

Effective prevention requires comprehensive cardiovascular risk management, public health initiatives for symptom awareness, CPR and AED training, and robust first responder networks. Early intervention is critical, as survival decreases by 7–10% with each minute of delay in defibrillation and CPR. Maintaining the chain of survival, early recognition, prompt CPR, rapid defibrillation, advanced life support, and integrated post-arrest care is vital to improving OHCA outcomes. A multifaceted approach combining risk factor control, community education, and emergency response capacity underpins reducing OHCA incidence and enhancing survival [4,9].

In an effort to maximize survivability, at least 18 European countries have implemented systems that incorporate first responders either fully or partially. In this context, first responders are defined as volunteers who are alerted to OHCA incidents by dispatch centers [10,11]. In 2016, following the completion of pilot studies, the Slovenian Resuscitation Council (SloRS) introduced a standardized licensing system for first responders nationwide, although this standard remains non-mandatory to this day [12]. SloRS followed the recommendation from the European Resuscitation Council (ERC) Guidelines on annual retraining to determine the recertification period. The current ERC Guidelines suggest retraining intervals ranging from a few months to one year, while clearly stating that there is no definitive research data available and that additional research is necessary. Neither the guidelines nor the SloRS recommendation clarifies whether a first responder who has missed at least one recertification needs to retake the basic course or if completing the recertification course alone is sufficient [13].

Several previous studies involving laypersons and non-healthcare professionals have demonstrated that cardiopulmonary resuscitation knowledge and practical skills may deteriorate within months after initial training, particularly when participants are not exposed to regular repetition or real-life resuscitation events [14,15]. Previous research has also suggested that repeated retraining, shorter retraining intervals, and distributed education approaches may improve long-term retention of CPR competencies, although findings remain heterogeneous and optimal retraining strategies are still uncertain [16]. According to the ERC Guidelines 2021, the educational objectives for first responders encompass mastering effective chest compressions and the safe operation of an AED, as well as acquiring ventilation skills in pediatric basic life support (BLS) [13]. In Slovenia, the basic course for licensed first responders (LFRs) also includes training on handling airway obstructions caused by foreign objects and managing significant external bleeding. The advanced module further educates participants on responding to recent stroke, chest pain, and introduces them to oxygen delivery methods and ventilation techniques using more sophisticated ventilation equipment [17]. The majority of LFRs currently consist of volunteer firefighters, as they are already equipped with pagers for receiving alerts and have insurance coverage for injuries or liability [12].

Although first responder systems are increasingly integrated into out-of-hospital cardiac arrest response across Europe and internationally, evidence regarding the long-term retention of practical skills and theoretical knowledge among licensed first responders remains limited [18]. The current European Resuscitation Council recommendations acknowledge the importance of regular retraining but do not define an evidence-based optimal recertification interval for this specific population, largely due to the lack of longitudinal studies evaluating the retention of competencies over time [13]. Existing research has predominantly focused on laypersons or healthcare professionals, while licensed first responders represent a distinct semi-professional group operating within organized emergency response systems. Furthermore, little is known about the influence of real-life intervention exposure on the maintenance of resuscitation competencies between certification periods [19]. Consequently, uncertainty persists regarding whether uniform time-based retraining strategies are sufficient for all first responders or whether recertification approaches should be adapted according to responder experience and intervention frequency [20,21].

Our study aimed to assess whether LFRs maintain a satisfactory level of theoretical knowledge and practical skills in CPR by the end of their license validity period. To this end, we evaluated newly licensed LFRs prior to their first recertification, as well as a cohort of more experienced LFRs. Additionally, we investigated the impact of the number of interventions during the certification period on the retention of knowledge and skills. By gaining a deeper insight into how first responders retain knowledge and skills, we can enhance the educational framework for first responders in Slovenia and beyond.

## 2. Materials and Methods

Our study started in 2019, and the last questionnaire from the initial certification courses was returned in 2024. A total of 534 sets of questionnaires for the initial certification courses were distributed to prehospital units throughout Slovenia during this five-year period. Before the recertification courses took place, additional batches of questionnaires were distributed at the end of the initial certificate validity period, according to the expected number of recertification candidates—another 400 sets. The last recertification questionnaires included in the present research were returned in early 2025.

### 2.1. Goals

Our primary research goal was to determine the differences in the retention of resuscitation knowledge and skills in a certain period after the initial certification among new and senior LFRs. Additionally, we examined the impact of the number of interventions between certifications on the retention of knowledge and skills.

### 2.2. Research Design

Research was conducted by experts consisting of emergency medicine specialists in 8 prehospital units on 26 volunteer fire departments nationwide. Participants were recruited from LFR certification and recertification courses conducted by participating prehospital units and volunteer fire departments. Newly certified LFRs were eligible if they completed the initial certification course and the immediate post-course assessment. Those newly certified LFRs were invited for reassessment before their first annual recertification. Senior LFRs were eligible if they were active LFRs with at least five years of experience following initial certification and attended annual recertification during the study period. Participants missing key practical skill and theoretical knowledge data were excluded from the corresponding analyses. We used an observational research strategy with a longitudinal time frame for newly certified LFRs and a cross-sectional comparison for the senior LFR cohort. Firstly, we educated the cohort of candidates for the newly certified LFRs and tested them immediately after the training process to establish their peak knowledge and skill level on the topic. A formally established education and certification procedure for LFRs in Slovenia was used. As the SloRS recommendation suggests, LFRs are issued a license for one year after they successfully finish a 10 h course on CPR with AED utilization, according to the current ERC Guidelines, as well as the guidelines on the control of important external bleeding and foreign body airway obstruction set by the Rules on Emergency Medical Service published in the *Official Journal of the Republic of Slovenia* [17]. Satisfactory levels of knowledge and skills are demonstrated by passing both the theoretical test and the practical examination, as recommended by the Slovenian Extended Professional College for Emergency Medicine. Annual recertification, composed of a minimum 5 h refresher course, a theoretical test, and a practical examination, is needed [6,17].

Newly certified LFRs were re-evaluated for skill and knowledge retention after one year. Re-evaluation took place before the first recertification. Additionally, in 2020, 2023, and 2024, we tested a cohort of senior LFRs, which are defined as LFRs who are still active and have at least 5 years of experience following initial certification. We tested them before their yearly recertification to compare their skill and knowledge retention with that of the newly certified LFRs. All newly certified LFRs that were tested immediately after initial certification were included. This group represents inexperienced LFRs who possess a satisfactory level of skill and theoretical knowledge, as required by the regulations. At re-evaluation, additional data regarding the number of OHCA interventions attended between the previous certification and recertification were collected. Only activations involving suspected or confirmed OHCA cases within the official first responder dispatch system were considered interventions for the purpose of this study. Intervention frequency was initially self-reported by participants and subsequently verified by authorized healthcare professionals with access to official intervention and dispatch records. Due to personal data protection regulations, the research team did not have direct access to identifiable intervention documentation.

Practical resuscitation skills were assessed using a modified Cardiff Test (Cardiff-19), based on the previously published Cardiff assessment tool for CPR and AED competency evaluation [22]. The assessment consisted of 19 items representing individual procedural steps within the BLS and AED algorithm and utilized a structured scoring system based on correctness, usefulness, and non-harmfulness of performance. The maximum achievable score was 55 points.

The original assessment tool was slightly modified to align with the current ERC Guidelines and the officially prescribed Slovenian LFR certification procedure. Modifications primarily included shortening the practical scenario to one CPR cycle, consisting of chest compressions and ventilations in a 30:2 ratio (approximately 2 min), followed by AED utilization, as well as separate coding of excessively high or low chest compression hand placement for observational purposes, while maintaining identical scoring values for incorrect placement. No substantial conceptual changes to the assessment structure were introduced.

All practical assessments were performed by experienced emergency medicine specialists routinely involved in LFR education and certification. Evaluators followed standardized assessment criteria and scoring instructions used nationally within the Slovenian LFR training system. Inter-rater consistency of the modified assessment was additionally evaluated during simultaneous independent scoring by two examiners and demonstrated no statistically significant differences between evaluators.

Data about theoretical knowledge were collected with a questionnaire composed of questions recommended by SloRS during planning of our preliminary research design [23]. It contains 9 mostly factual questions, each with one correct answer out of 4 available options, and one question with a number scale to mark the correct answer. The total possible points that can be achieved is 10, with 0 points being the lowest number [23]. To ensure data comparability, we employed identical questions across all certifications. We implemented measures such as prohibiting the photographing of the questionnaire, taking separate notes on paper, and using electronic devices during examinations; moreover, at least two examiners were required to proctor the examination to mitigate the risk of exam misconduct or the dissemination of content to future cohorts. Given that the questions were predominantly basic and factual, potential pre-exposure to the questions was not considered a disadvantage, since studies indicate that pre-exposure to factual questions may enhance learning efficiency, promote longer knowledge retention, and does not lead to biased results [24,25,26,27,28].

We also examined how the number of OHCA interventions a first responder had participated in following the last certification affected the retention of resuscitation knowledge and skills across all the previously mentioned groups. Prior to performing a statistical analysis, the newly certified LFRs were categorized based on interventions into three groups: 1, no interventions; 2, one intervention; and 3, two or more interventions after initial certification. Similarly, we categorized senior LFRs into two groups: (1) no interventions and (2) one or more interventions after the last recertification.

No formal a priori sample size calculation was performed because the study was based on the availability of participants attending nationally organized certification and recertification courses during the study period. The final sample size, therefore, reflected the number of eligible LFRs available for assessment and follow-up within participating centers.

### 2.3. Data Analysis

A database was established for analysis and subsequently imported into the statistical analysis software Jamovi v2.6.26. A *p*-value of less than 0.05 was considered to indicate statistical significance, where applicable.

Cases with missing practical skill or theoretical knowledge outcome data were excluded from the corresponding statistical analyses using complete-case analysis. Participants with partially incomplete datasets were included in analyses where all required variables were available. The proportion of missing data was low and was primarily related to technical issues, participant absence at recertification, or incomplete questionnaire return.

The Shapiro–Wilk test was employed to assess the normality of the distribution. To investigate the retention of resuscitation skills and theoretical knowledge by newly certified LFRs until the day of their first recertification, descriptive statistics analysis was performed. The non-parametric Wilcoxon signed-rank test was used when the distribution was non-normal; otherwise, the parametric paired sample *t*-test was used. To compare the skills and knowledge of senior LFRs with those of newly certified LFRs, an independent sample statistical analysis was required. If the Shapiro–Wilk test indicated that the data were not normally distributed, we used the Mann–Whitney U-test; otherwise, we employed the independent sample *t*-test. Effect sizes were reported as the rank biserial correlation (rbc) for non-parametric analyses and Cohen’s d for parametric analyses.

In addition, we conducted a linear regression analysis to examine the differences in skills and knowledge concerning the number of OHCA interventions for newly certified LFRs. A similar linear regression analysis was performed within the group of senior LFRs. However, due to the lack of data on their initial skills and knowledge scores, our focus was on the relationship between skills and knowledge scores and the number of OHCA interventions. Given that the data on the number of OHCA interventions was collected as ordinal categorical variables, correlation analysis was deemed inappropriate.

## 3. Results

Of the 534 questionnaires distributed during the initial courses, 342 were completed and returned, yielding a response rate of 64%. An additional 400 questionnaires were distributed for assessments conducted prior to the first recertification. A total of 125 questionnaires were returned, representing a 37% return rate. Cases with missing data on both skill and knowledge performance were excluded by default (28 cases; 22%). Our team successfully re-evaluated 97 LFR participants after a one-year interval following their initial certification. Additionally, 140 senior LFR participants were assessed prior to their regular recertification to compare their resuscitation skills and theoretical knowledge with those of newly certified LFRs. Most of the returned questionnaire sets were fully completed, with only a small proportion containing missing data or incomplete responses. Supplementary File S1 includes a diagram illustrating participant flow and the number of questionnaires available for each data analysis.

### 3.1. Newly Certified LFRs’ Skill and Knowledge Retention

Newly certified LFRs demonstrated similar skill retention scores immediately following the initial course compared to assessments conducted prior to the first re-evaluation (N = 97). We were unable to demonstrate statistically significant differences in theoretical knowledge retention scores between the assessments conducted after the initial course and the first re-evaluation (N = 94). The results are presented in Table 1.

### 3.2. Senior LFRs’ Skill and Knowledge Performance

Practical skill scores among senior LFR participants prior to annual recertification were significantly lower than in the newly certified LFR group (N = 131 vs. 337). In contrast, theoretical knowledge scores among senior LFRs did not differ significantly from the newly certified LFRs group (N = 131 vs. 333), as shown in Table 1.

### 3.3. Impact of Participating in OHCA Interventions on Skills and Theoretical Knowledge Retention

We conducted a statistical analysis of the groups formed based on the number of interventions they participated in following their initial certification. The analysis among newly certified LFRs revealed no statistically significant difference in skill retention among the three groups: those without intervention (N = 39); those with one intervention (N = 17); and those with two or more interventions (N = 37). No significant difference was also found in theoretical knowledge retention in any of the groups. The results are gathered in Table 2. Among the newly certified LFRs, intervention frequency did not significantly predict changes in either practical skill retention or theoretical knowledge scores. Compared with LFRs without interventions, participation in one intervention (β = −0.796, *p* = 0.570) or two or more interventions (β = 0.183, *p* = 0.868) did not significantly predict changes in practical skill scores (R^2^ = 0.006). Similarly, participation in one intervention (β = 0.297, *p* = 0.456) or two or more interventions (β = 0.396, *p* = 0.210) was not significantly associated with changes in theoretical knowledge scores (R^2^ = 0.018).

Senior LFRs were also divided into two groups: those without intervention and those with at least one intervention after the last certification. Their skills and theoretical knowledge were compared to newly certified LFRs. The group without intervention (N = 28) demonstrated significantly inferior performance in skills, but no difference in theoretical knowledge scores. There were no statistically significant differences in skill or knowledge between LFRs who participated in interventions (N = 102) and newly certified LFRs following initial certification. The results are represented in Table 3. Among the senior LFRs, participation in at least one intervention significantly predicted higher practical skill scores (β = −1.11, *p* = 0.001; R^2^ = 0.024), while no significant association was found between intervention participation and theoretical knowledge retention (β = −0.270, *p* = 0.070; R^2^ = 0.008). To facilitate a clearer understanding of the practical research findings, a summary is provided in Table 4.

## 4. Discussion

This study contributes to the limited body of evidence regarding long-term retention of basic life support competencies among licensed first responders operating within organized community response systems. While current European Resuscitation Council recommendations acknowledge the importance of regular retraining, the optimal recertification interval and the influence of real-life intervention exposure remain insufficiently defined. Our findings demonstrate that newly certified first responders can maintain practical skills and theoretical knowledge during the first certification year, whereas among experienced responders, participation in real-life interventions appears to be associated with better practical performance. These findings may have implications not only for Slovenia, but also for other European and international first responder systems that rely on volunteer or semi-professional responders integrated into emergency medical dispatch networks [29]. The results support the concept that retraining strategies could be adapted according to responder experience and real-life exposure rather than relying exclusively on uniform time-based recertification intervals [30,31,32,33].

The present findings suggest that the current annual retraining recommendation may be adequate during the first year following certification. No significant decline in skills or knowledge was observed among newly certified LFRs prior to their first annual recertification, irrespective of the number of OHCA interventions they participated in. Conversely, senior LFRs demonstrated lower practical skill performance compared with newly certified LFRs, whereas their knowledge performance remained comparable. Lower practical skill performance among senior LFRs was primarily observed in those who had not participated in any OHCA interventions during the certification period. Senior LFRs who had participated in at least one OHCA intervention demonstrated practical performance comparable to the newly certified LFRs.

### 4.1. Retention of Skill and Knowledge

Extensive debate and research have been conducted regarding the training and retraining of laypersons as potential bystanders or passively alerted non-professionals in the event of an OHCA incident in their vicinity [9,34]. The retraining period for these groups remains inadequately defined. Consequently, the ERC Guidelines 2021 acknowledge the importance of regular retraining; however, the optimal recertification interval remains insufficiently defined and is still largely based on expert opinion rather than robust longitudinal evidence [13]. SloRS followed this recommendation in their own recommended protocols [23]. Furthermore, as in many other countries, LFRs in Slovenia occupy a unique position that does not align precisely with the categories of healthcare professionals or complete laypersons [35,36]. They are integrated into the official alert system, their interventions are recognized as official, and they hold licenses that require annual renewal through a recertification course. Although the majority of LFRs participate on a voluntary basis, their involvement is considered part of the operations of emergency protection, rescue, and relief services. This situates them in a semi-professional category, for which no specific retraining period is currently delineated [6,13]. Due to the non-mandatory nature of these recommendations and their lack of robust empirical foundations, some educators in Slovenia do not follow the annual retraining and curriculum recommendations. As a result, Slovenia employs various retraining time periods and levels of retraining comprehensiveness. Our research initiated an investigation into this issue by assessing whether the recommended annual retraining is sufficient for LFRs’ minimal skill and knowledge retention. The results indicated that newly certified LFRs do not exhibit any decline in skills and theoretical knowledge after one year. In contrast, senior LFRs showed lower practical skill performance compared with newly certified LFRs, but their theoretical knowledge performance was comparable. As indicated in past research, a different approach might be needed for more experienced participants [37].

### 4.2. Impact of the Number of OHCA Interventions

Current research on skill retention among healthcare professionals consistently indicates a rapid decline in CPR competencies following training [38]. In contrast, recent investigations into emergency medicine (EM) physicians, who frequently manage cardiac arrest cases, suggest that supplementary BLS retraining does not enhance their CPR performance [39]. The diverse spectrum of healthcare professions leads to considerable variation in the frequency with which professionals encounter CPR scenarios. These findings suggest that the frequency of real-life CPR interventions may be associated with better skill retention among healthcare workers [38,39]. These findings may also be relevant in the broader context of OHCA outcomes. Previous research has demonstrated that early, effective bystander and first-responder interventions are strongly associated with improved survival and neurological outcomes after OHCA [40]. In this regard, the present findings suggest that maintaining practical competencies among first responders, particularly through regular real-life intervention exposure, may contribute to sustaining the quality of early resuscitation efforts within community response systems. Similarly, our research corroborated that for more experienced LFRs, actual interventions appeared to contribute to achieving skill levels comparable to newly certified LFRs. This interpretation is additionally supported by linear regression analysis, which identified participation in at least one real-life intervention as a significant predictor of higher practical skill performance among senior LFRs. No comparable association was observed for theoretical knowledge performance or within the cohort of newly certified LFRs, suggesting that practical experience may contribute to maintaining psychomotor competencies among experienced responders. Senior LFRs who did not participate in interventions demonstrated lower BLS practical skill performance compared with newly certified LFRs prior to annual recertification, in contrast to their peers who engaged in interventions. This could be attributed to the fact that recertification training is typically shorter than initial certification, or it might be due to their altered perspective and attitude after many years of involvement. This suggests that an alternative strategy for the retraining of experienced LFRs may be necessary, such as through the method of learning by teaching [37]. Nevertheless, their theoretical knowledge scores remained comparable to those of the newly certified LFRs prior to recertification. Notably, among the newly certified LFRs, there was no observed decline in skills or knowledge, regardless of their participation in interventions. This finding confirms the adequacy of initial LFR training in Slovenia and first recertification after one year, particularly when compared with studies reporting lower CPR skill performance after similar or shorter retraining intervals [41].

### 4.3. Limitations

Firstly, due to the COVID-19 pandemic in 2020 and 2021, we were unable to test any LFRs or provide them with resuscitation skills or knowledge repetition during that period, as daily migrations outside the municipality were restricted without a highly excusable reason. In addition, all activities that could facilitate aerosol transmission of the virus, including even LFR activations for a brief period, were prohibited. Thus, from mid-2020 to early 2022, we did not collect any data. This is also the reason we had to extend our research to obtain at least a minimal number of cases, ensuring the research is representative. Participants initially tested in 2019 and 2020 could only be included in the general LFR population, representing the peak practical performance and theoretical knowledge level after the first certification.

Secondly, in comparison to newly certified LFRs, most senior LFRs passed the initial certification course years before our research began; we did not have data on their peak performance in either practical or theoretical aspects after completing the initial course. We were only able to compare their present abilities with the average performance of newly certified LFRs at the end of the initial courses. Therefore, we could not analyze the data as paired samples, and thus had to use independent sample statistical analysis. Although intervention frequency was additionally verified by authorized healthcare personnel with access to dispatch documentation, the initial reporting relied on participant self-reporting and may, therefore, be subject to minor reporting inaccuracies.

Thirdly, expert examiners also reported various limitations that led to dropped cases; the most prevalent reasons were technical issues, LFRs not showing up for the recertification course, and a lack of time for additional work on the research protocols, among others. Furthermore, numerous newly certified LFRs successfully completed recertification in courses not conducted by our team of experts. These factors may also have introduced selection bias, as participants who completed recertification and agreed to participate may have been more motivated or educationally engaged than non-participants.

Finally, the organization, training structure, and operational role of LFRs in Slovenia may differ from first responder systems in other countries, which may limit the generalizability of the findings to other emergency response systems.

## 5. Conclusions

Our research did not identify any significant deterioration in the BLS skills and knowledge of newly certified LFRs after one year, regardless of the number of OHCA interventions. An extensive initial training program, therefore, appears to be sufficient during the first certification year.

Senior LFRs demonstrated lower BLS practical skill performance compared with newly certified LFRs prior to annual recertification. Lower practical performance was primarily observed among senior LFRs who had not engaged in any OHCA interventions since their last certification. Conversely, senior LFRs who participated in at least one intervention demonstrated practical skill performance comparable to newly certified LFRs. Theoretical knowledge performance remained comparable across all groups.

Given that the primary distinction between basic and refresher training sessions lies in their extensiveness, future retraining strategies for senior LFRs might potentially benefit from considering real-life intervention exposure. However, these findings should be interpreted cautiously, as the present study compared senior LFRs cross-sectionally with newly certified responders rather than through individual longitudinal follow-up. Further longitudinal studies are therefore needed to determine whether recertification intervals and training intensity should be adapted according to OHCA intervention frequency and responder experience, particularly among LFRs who have missed annual recertification or have not participated in BLS interventions for extended periods.

## Figures and Tables

**Table 1:** Retention of skill and knowledge among licensed first responders (LFRs).

Group	Skills		Knowledge	
Newly certified LFRs (Median [IQR]; *p*-value; effect size) *	53 [52–54] vs. 53 [50–54]	*p* = 0.059 rbc = 0.196	10 [9–10] vs. 9 [9–10]	*p* = 0.458 rbc = 0.017
Senior LFRs (Median [IQR]; *p*-value; effect size) **	51 [49–54] vs. 54 [52–55]	*p* < 0.001 rbc = −0.349	9 [8.5–10] vs. 10 [9–10]	*p* = 0.091 rbc = 0.073

* Compared to their own results on the initial certification a year prior (Wilcoxon signed-rank test). ** Compared to the results of the LFRs on the initial certifications (Mann–Whitney U-test). Abbreviations: LFRs, licensed first responders; IQR, interquartile range; and rbc, rank biserial correlation.

**Table 2:** The impact of the number of interventions on the retention of skills and knowledge among newly certified LFRs compared to their own results on the initial certification a year prior.

Number of OHCA Interventions	Newly Certified LFRs			
	Skills		Knowledge	
No intervention (Median [IQR]; *p*-value; effect size)	53 [50–54] vs. 52 [48–53]	*p* = 0.135 rbc = 0.171	10 [9–10] vs. 9 [9–10]	*p* = 0.211 rbc = 0.205
1 (Median [IQR]; *p*-value; effect size)	53 [52–55] vs. 52 [51–55]	*p* = 0.095 rbc = 0.203	8.53 ± 1.23 vs. 8.65 ± 1.27 *	*p* = 0.605 Cohen’s d = 0.065
2 or more (Median [IQR]; *p*-value; effect size)	52.5 ± 4.31 vs. 52.1 ± 3.63 *	*p* = 0.169 Cohen’s d = 0.071	10 [9–10] vs. 10 [9–10]	*p* = 0.842 rbc = 0.025

For statistical analysis, the Wilcoxon signed-rank test was used. For the effect size, the rank biserial correlation (rbc) is presented. * Due to the data being normally distributed, the paired sample *t*-test was used. The results are presented as mean ± SD; the effect size is presented as Cohen’s d.

**Table 3:** The impact of the number of OHCA interventions on the retention of skills and knowledge among senior LFRs compared to the results from the initial LFR certifications.

Number of OHCA Interventions	Senior LFRs			
	Skills		Knowledge	
No intervention (Median [IQR]; *p*-value; effect size)	49 [47–51] vs. 54 [52–55]	*p* < 0.001 rbc = −0.237	9.5 [8–10] vs. 10 [9–10]	*p* = 0.439 rbc = −0.080
1 or more (Median [IQR]; *p*-value; effect size)	52 [50–54] vs. 54 [52–55]	*p* = 0.117 rbc = −0.082	9 [9–10] vs. 10 [9–10]	*p* = 0.102 rbc = 0.076

For statistical analysis, the Mann–Whitney U-test was used.

**Table 4:** Practical interpretation of skill and knowledge retention outcomes among LFRs.

Group	Practical Skills After 1 Year *	Theoretical Knowledge After 1 Year *	Impact of OHCA Intervention Participation **
Newly certified LFRs	Maintained	Maintained	No significant association observed
Senior LFRs overall	Lower performance compared with newly certified LFRs	Comparable to newly certified LFRs	Participation is associated with better practical performance
Senior LFRs without interventions	Lowest performance	Comparable to newly certified LFRs	Non-participation is associated with poorer practical performance
Senior LFRs with ≥1 intervention	Comparable to newly certified LFRs	Comparable to newly certified LFRs	Better practical performance than non-participants

* Based on longitudinal analysis (newly certified LFRs) and cross-sectional comparison (senior LFRs). ** Based on linear regression findings.

## Data Availability

The datasets presented in this article are not readily available because the data are part of an ongoing study and due to General Data Protection Regulation rules.

## Supplementary Materials

The following supporting information can be downloaded at: https://www.mdpi.com/article/10.3390/healthcare14121654/s1. Supplementary File S1: Diagram of participant flow and number of questionnaires with data availability for analysis.
