# Supplementary material for: Preservation of Basic Life Support Competencies Among Certified First Responders
Source: Healthcare (Basel). 2026 Jun 11;14(12):1654. doi: 10.3390/healthcare14121654 (PMC13300716; doi:10.3390/healthcare14121654)
Supplement: Supplementary file 1 [file healthcare-14-01654-s001.zip › healthcare-4265160-supplementary.pdf]

Supplement File S1

Diagram of participant flow and number of questionnaires with data availability for analysis

<u>NEW LFRs</u>

Initial course:

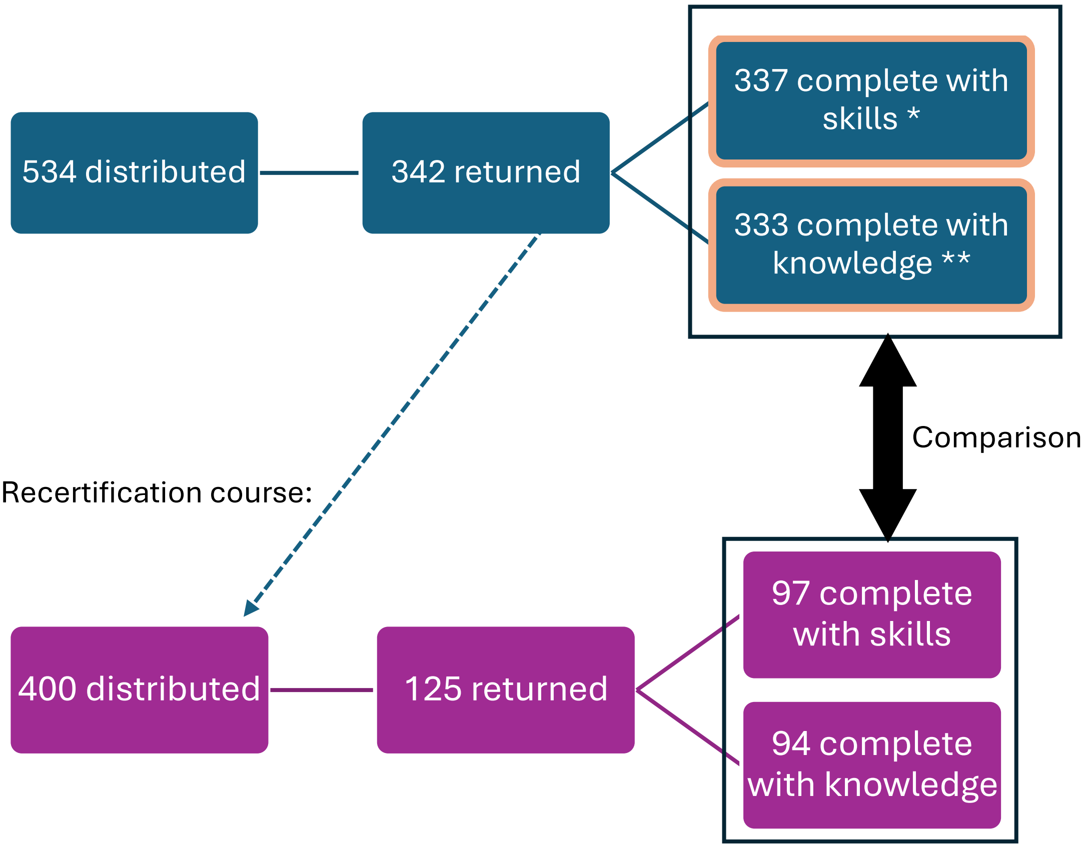

<u>SENIOR LFRs</u>

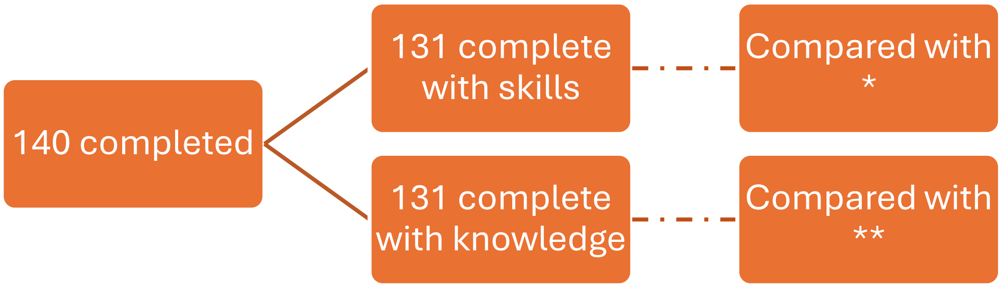

IMPACT OF NUMBER OF INTERVENTIONS

New LFRs:

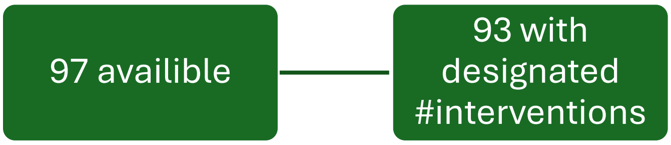

Senior LFRs:

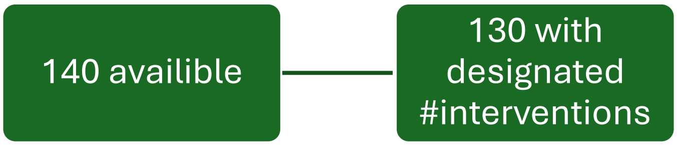

List of abbreviations:

LFRs – *Licensed First Responders*
